# Development of Antimicrobial Comb-like Hydrogel Based on PEG and HEMA by Gamma Radiation for Biomedical Use

**DOI:** 10.3390/gels12010032

**Published:** 2025-12-30

**Authors:** Alfredo Contreras, Alejandra Ortega, Héctor Magaña, Jonathan López, Guillermina Burillo

**Affiliations:** 1 Instituto de Ciencias Nucleares, Departamento de Química de Radiaciones y Radioquímica, Universidad Nacional Autónoma de México, Mexico City 04510, Mexico; 2 Facultad de Ciencias Químicas e Ingeniería, Universidad Autónoma de Baja California, Parque Industrial Internacional Tijuana, Avenue Universidad 14418, Tijuana 22390, Mexico; hector.magana@uabc.edu.mx (H.M.);

**Keywords:** antimicrobial material, comb-like hydrogel, gamma radiation, ciprofloxacin delivery, PEG, HEMA

## Abstract

Poly(ethylene glycol) (PEG) and poly(2-hydroxy ethyl methacrylate) are polymers used for many biomedical applications due to their biocompatibility, non-toxicity, and antibiofouling properties. In this work, a new comb-like hydrogel based on 2-hydroxyethyl methacrylate (HEMA) grafted onto a polyethylene glycol network (*net*-PEG) was synthesized by gamma radiation from Co^60^ in two steps. First, PEG (Mw = 20,000) was crosslinked at 30 kGy, and then HEMA was grafted, varying the concentration (5–20% *v*/*v*) and irradiation dose (2.5–15 kGy). Results of infrared spectroscopy (FTIR) and thermogravimetric analysis (TGA) confirmed the incorporation of HEMA onto *net*-PEG. Moreover, the properties of comb-like hydrogel (*net*-PEG)-*g*-HEMA were studied through swelling kinetics, drug loading and release, antimicrobial activity, and biocompatibility assays. The findings showed a different behavior in swelling kinetics and drug delivery depending on HEMA grafting. Comb-like hydrogel with 30 and 66% grafting could load more ciprofloxacin (2 mg g^−1^) than *net*-PEG (1.5 mg g^−1^) but only release 38 and 48% at 24 h, respectively. In addition, all drug-loaded hydrogels displayed inhibition for Gram-negative bacteria (*E. coli*) and a cell viability superior of 95% using mouse embryonic fibroblasts (BALT/T3). Comb-like hydrogel has potential application in the biomedical field such as in wound dressings or controlled drug delivery systems.

## 1. Introduction

Hydrogels are three-dimensional networks composed of hydrophilic polymers capable of retaining a large amount of water or biological fluids without dissolving. They have gained significant importance in recent decades due to their high water absorption which produces a soft and porous material like natural tissues, mimicking the extracellular matrix [1,2]. Currently, they have been successfully used in various biomedical applications depending on their physicochemical, biological, and structural properties [3], standing out as scaffolds, wound dressings, and drug delivery devices [4]. Nevertheless, not all hydrogels possess biocompatibility and biodegradability, which can be adjusted with the appropriate choice of polymers and the synthesis method.

In this context, poly(ethylene glycol) (PEG) and poly(2-hydroxyethyl methacrylate) (PHEMA) are widely used in biomedical applications due to their biocompatibility, non-toxicity, and antibiofouling properties [5,6,7]. PEG hydrogel is known for its flexibility and ease of modification [8], while HEMA hydrogel offers good mechanical properties and chemical stability [9]. Combining these materials has allowed the obtention of hydrogels with improved properties such as mechanical strength, thermal stability, chemical resistance, and porosity, among others [4,9,10]. However, most of the PEG-HEMA hydrogels reported in the literature have been obtained by chemical [10,11,12,13], UV radiation [14,15,16], and microwave methods [17,18,19], presenting some disadvantages such as complicated methodologies of synthesis and purification [10,16], long reaction time [9,10,12], and high temperatures [11,12,13,14]. For example, Son et al. synthesized hydrogels based on HEMA and poly(ethylene glycol) methyl ether methacrylate (PEGMEMA) through a chemical method using ammonium persulfate (APS) as a redox initiator at 45 °C for 20 h. Although incorporation of PEG enhanced the porosity and hydrophilicity of PHEMA hydrogel, the methodology implies various steps making its purification difficult [10]. Hu and coworkers designed a semi-interpenetrating network of PHEMA, 2-methacryloyloxyethyltrimethyl ammonium chloride (METAC), and PEG, showing that the swelling ratio was better than that of the HEMA network. In addition, an in vitro cytotoxicity study showed good cytocompatibility, but high temperatures (60–85 °C) were required for the crosslinking process [12]. Bayramoglu et al. studied hydrogels in film form using PHEMA, poly(ethyleneglycol) methacrylate (PEGMA), and hydroxypropyl chitosan by UV photopolymerization for seeding mesenchymal cells. The hydrogels showed poor ability to form colonies compared to pristine PHEMA, and an extensive purification of material by a Soxhlet system was required for its subsequent use as a scaffold [16]. Pisheh et al. prepared hydrogels of PEG and HEMA for regenerative medicine, obtaining a material with good mechanical properties and low cytotoxicity. However, the synthesis required first the obtention of the vinyl ester macromonomer (HEMA-TA-PEG) by microwaves, followed by the crosslinking by chemical treatment at 80 °C using 3-glyciloxypropyl trimethoxysilane, APS, and benzoic peroxide [17]. An effective alternative to the aforementioned methods is gamma radiation because it offers advantages such as simplicity, reproducibility, efficiency, and high quality of the materials obtained [20,21]. Gamma rays are high-energy photons with high penetrating power that produce mainly free radicals when interacting with matter. These induce various chemical reactions (chain scission, grafting, and crosslinking) without the use of chemical compounds (initiators, catalysts, or crosslinkers), facilitating the purification of the materials [21]. Furthermore, radiation crosslinking is more homogeneous, allowing better control of the size and distribution of the networks, which can affect the hydrogel properties and performance [22].

The novelty of this work is the synthesis of a comb-like hydrogel composed of a polyethylene glycol network (*net*-PEG) and 2-hydroxyethyl methacrylate (HEMA) using only gamma radiation with potential biomedical uses. A comb-like hydrogel, consisting of a crosslinked polymeric backbone grafted with side chains, exhibits unique physicochemical behaviors compared to other hydrogel architectures (semi-interpenetrated, interpenetrated, blends, etc.), highlighting faster responsiveness and transport properties [23,24,25]. These hydrogels are ideal materials for drug delivery or biomolecule diffusion due to swelling and deswelling kinetics being improved, which can be tuned based on the amount of grafted side chains [23,26]. Thus, in the first step, PEG was crosslinked (*net*-PEG) by varying irradiation dose and polymer concentration. Subsequently, *net*-PEG was grafted with HEMA in a second step by gamma radiation to evaluate its properties as a function of the graft. The incorporation of HEMA onto *net*-PEG was confirmed by infrared spectroscopy (FTIR) and thermogravimetric analysis (TGA). Comb-like hydrogel (*net*-PEG)-*g*-HEMA was studied through swelling behavior, ciprofloxacin loading and release kinetics, antimicrobial activity, and biocompatibility assays. Results show that hydrogel has potential applications in the biomedical field such as in controlled drug delivery systems and antimicrobial material.

## 2. Results and Discussion

### 2.1. Obtention of net-PEG

**Figure 1:**
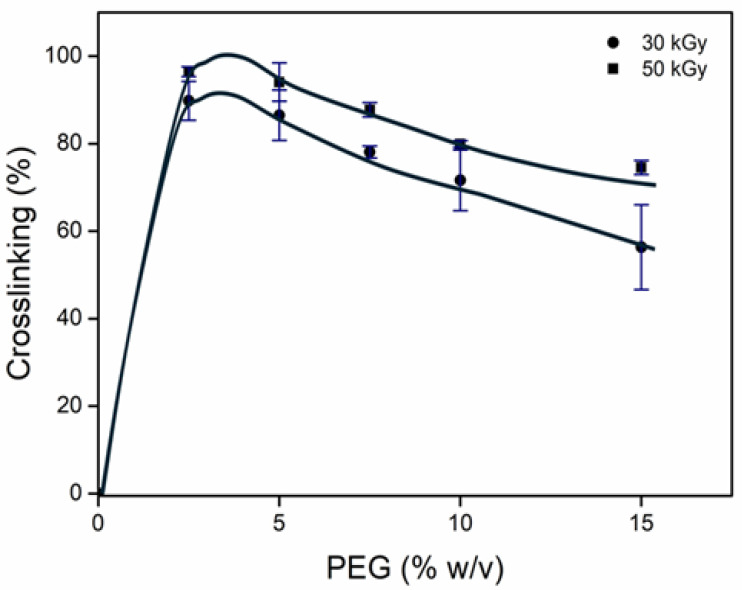
Crosslinking percentage as a function of PEG concentration and dose. *n* = 4.

PEG crosslinking was performed by gamma radiation, producing free radicals on PEG chains that react with other polymer chains to link them through covalent bonds and create a three-dimensional network [27]. Crosslinking performance was studied by varying the dose and PEG concentration as shown in Figure 1.

Maximum yield was achieved at low PEG concentrations, and then it gradually decreased with higher concentrations. This behavior was equal for both tested doses. Crosslinking yield (90%) was similar between 2.5 and 5% *w*/*v* PEG, but the amount of material obtained was greater when PEG concentration was high. On the other hand, when the dose was varied from 30 to 50 kGy, the crosslinking yield increased slightly (<10%) although not significantly considering that the irradiation dose was almost double. This happens because the viscosity of PEG solution increases proportionally with concentration, causing lower mobility of the macroradicals and hindering the reticulation process [27]. For these reasons, *net*-PEG for the synthesis of comb-like hydrogel was made using a PEG concentration of 5% *w*/*v* and a dose of 30 kGy.

### 2.2. Obtention of Comb-like Hydrogel: (net-PEG)-g-HEMA

**Scheme 1:**
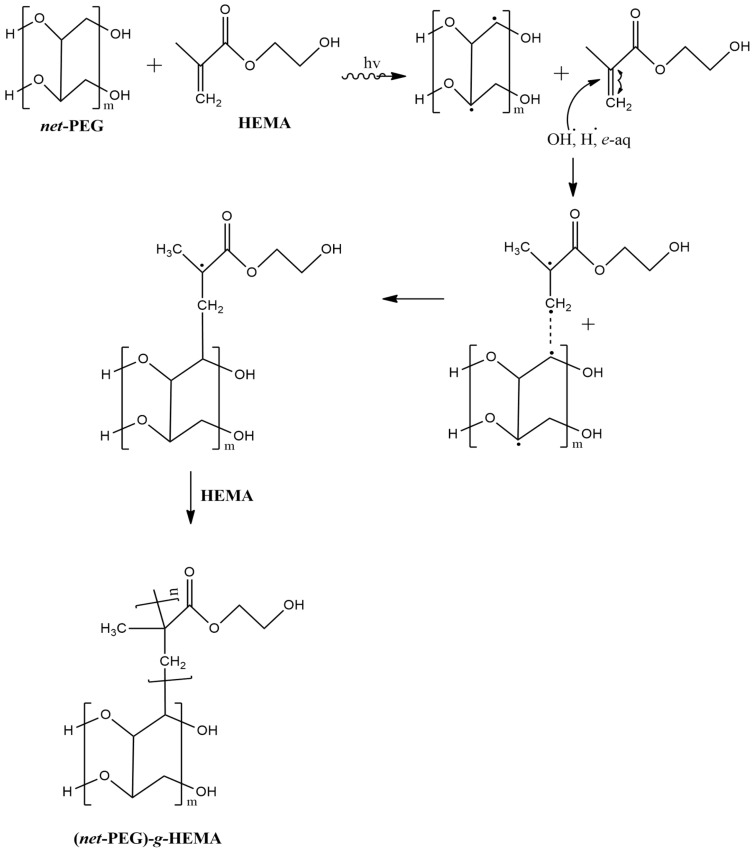
Mechanism of synthesis of comb-like hydrogel based on *net*-PEG and HEMA by gamma radiation.

PEG hydrogel was grafted with HEMA, varying the dose and HEMA concentration to evaluate the impact of these variables on material performance, which can modify the hydrophilicity, morphology, and antimicrobial properties of the comb-like hydrogel. Synthesis was carried out by a direct method where all compounds are irradiated together. The grafting mechanism implies the homolytic opening of the C=C bond of the methacrylate moiety of HEMA by the free radicals produced when gamma radiation interacts with the matter, with water radiolysis being the main mechanism responsible for generating these species (*OH, *H, and ^−^e_aq_) [28]. HEMA radicals react with *net*-PEG macroradicals to initiate the grafting reaction, while at the same time HEMA homopolymerization occurs as a side reaction (Scheme 1).

**Figure 2:**
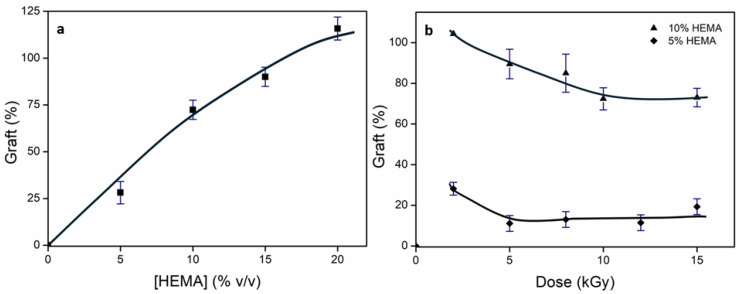
HEMA grafting onto *net*-PEG using ethanol/water (1:1) as solvent as a function of (**a**) HEMA concentration (10 kGy) and (**b**) dose. *n* = 6.

The graft kinetics results are shown in Figure 2a, where it can be noted that it increased quickly with concentration to achieve yields up to 100% at a HEMA concentration of 20% *v*/*v*. This happens because HEMA is a radiation-reactive monomer that polymerizes easily and even crosslinks if the concentration or irradiation dose increases [29]. Moreover, the viscosity of the system also increases with HEMA concentration, which diminishes the radical’s mobility and hinders the graft process [30,31]. These results indicate that it is better to work with HEMA concentrations of 5–10% *v*/*v* to obtain grafting less than 75%. On the other hand, the irradiation dose was also a crucial factor that affected the grafting yield. Figure 2b shows that the highest yield was obtained at 2.5 kGy and then decreased until reaching a plateau after 8 kGy. This behavior was displayed for both tested concentrations, indicating that radicals’ diffusion controls the graft process. At higher doses more radicals are generated, but at the same time the viscosity of the system increases, which impairs the grafting reaction due to the low mobility of the free radicals. Thus, it is possible to control graft percentages (10–100%) by varying the HEMA concentration (5 and 10% *v*/*v*) and irradiation dose (2.5–8 kGy).

### 2.3. Characterization

#### 2.3.1. FTIR and TGA

**Figure 3:**
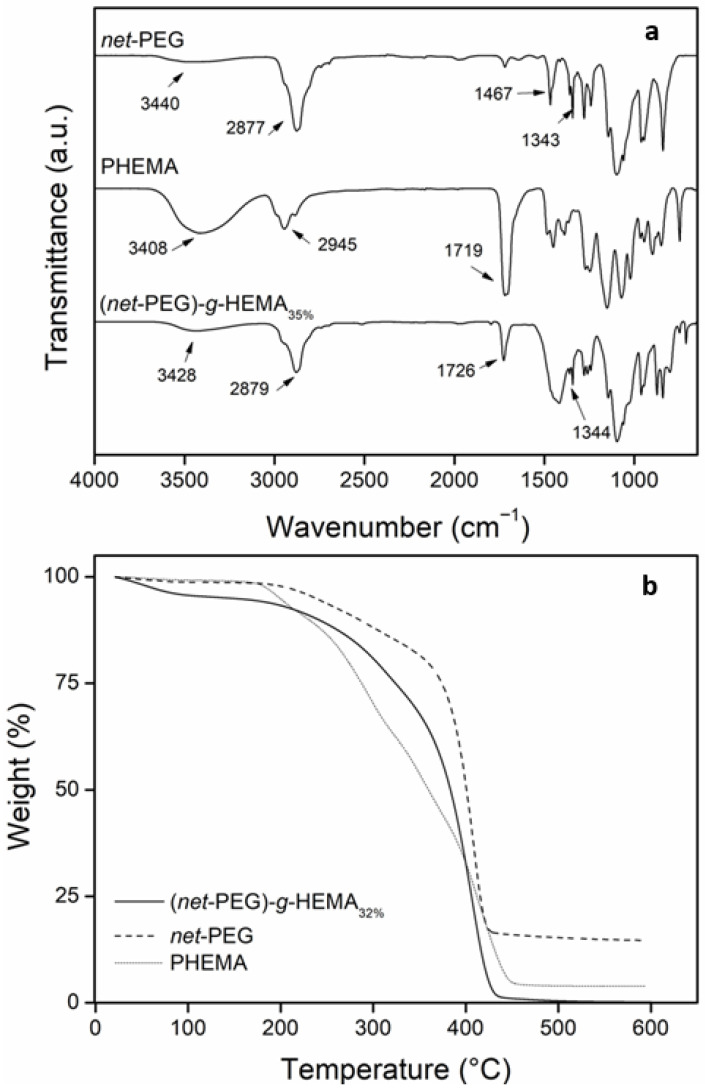
Characterization of *net*-PEG, PHEMA, and comb-like hydrogel for (**a**) FTIR and (**b**) TGA.

Hydrogels were analyzed by infrared spectroscopy and results are shown in Figure 3a. *net*-PEG displayed its characteristic absorption bands at 3440, 2877, 1467, and 1343 cm^−1^ corresponding to stretching and bending vibrations of hydroxyl (-OH) and methylene groups (-CH_2_) [32]. The PHEMA spectrum showed bands at 3408, 2945, and 1719 cm^−1^ associated with the hydroxyl (-OH stretching), methylene (-CH_2_ stretching), and ester groups (C=O stretching), respectively [32]. Comb-like hydrogel (35% graft) exhibited the absorption bands of both *net*-PEG and PHEMA, where the peak at 1726 cm^−1^ of ester carbonyl (C=O stretching) confirmed the incorporation of methacrylate compound since PEG hydrogel does not possess this functional group.

Thermal decomposition of materials is shown in Figure 3b. *net*-PEG exhibited two degradation steps at 248.5 and 408.5 °C associated with the cleavage of ethylene glycol moiety and the carbon chain, respectively. The mechanism involves the breaking of C-O and C-C bonds in the PEG backbone [33,34]. PHEMA showed one initial decomposition temperature around 195 °C and the process was completed at 450 °C. During thermal degradation, PHEMA breaks down into its monomer, along with ethylene glycol and methacrylic acid [35]. Comb-like hydrogel (32% graft) displayed loss of water (4.45%) and two decomposition stages at 308 and 407 °C corresponding to grafted HEMA and *net*-PEG, respectively. These findings demonstrate that comb-like hydrogel preserves good thermal stability since it begins its decomposition at 200 °C, which is a higher temperature than that used for autoclaves (120–130 °C). This is important for materials for biomedical applications, where they must be sterilized usually by this technique.

#### 2.3.2. Swelling Behavior

**Figure 4:**
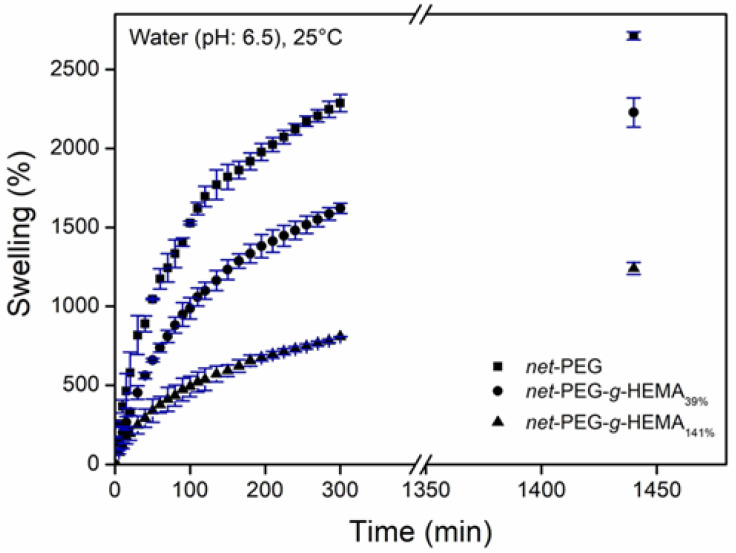
Swelling kinetics of *net*-PEG and comb-like hydrogel with different HEMA grafting percentages. *n* = 4.

Samples’ hydrophilicity was measured by water absorption studies at 25 °C in order to determine the swelling equilibrium and the time needed to achieve it (Figure 4). Results showed that *net*-PEG was able to retain a large amount of water (2600%) at 24 h since water molecules interact with the hydroxyl groups of PEG chains through hydrogen bonds [36]. Comb-like hydrogel (*net*-PEG)-*g*-HEMA displayed less swelling and it was proportional to HEMA graft.

Due to multiple factors, when *net*-PEG is grafted with HEMA in a second step, it receives more radiation that increases the amount of covalent bonds between polymeric chains (crosslinking density), which has been widely reported in the literature [20,21,22,23]. This means that the network has a more compact structure, reducing the pore size and the swelling capacity [37]. In addition, the HEMA graft further reduces the internal space of the hydrogel, also limiting the amount of water it can retain [38]. It is well known that swelling of polymeric networks depends on their microscopic structure, specifically their orientation, entanglement, and the nature of the connections between them [38,39]. The structural elements directly impact on the amount of solvent that a polymer network can absorb and how it deforms during the swelling process. Thus, comb-like hydrogel with 39 and 141% grafting exhibited swelling percentages of 2100 and 1800% at 24 h, respectively. Furthermore, the equilibrium swelling time was 24 h for all hydrogels, indicating that it was independent of HEMA graft.

These results are good for our purposes because (*net*-PEG)-*g*-HEMA still retains a large amount of water and can be handled without breaking. This is possible because the mechanical properties can be improved with grafting, as the entanglement between the molecular chains increases, promoting better dispersion of the components and creating a more uniform structure [40].

#### 2.3.3. In Vitro Drug Loading and Release Studies

The PEG network and comb-like hydrogel with different graft percentages (30 and 66%) were loaded with ciprofloxacin in order to provide them with antimicrobial activity. This drug is a broad-spectrum antibiotic of the fluoroquinolone class and one of the most widely used to treat infections because of its efficacy, safety, and relatively low cost [41].

**Figure 5:**
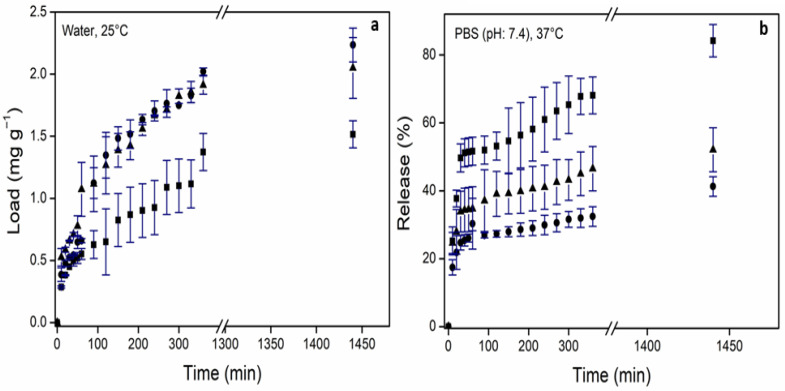
(**a**) Load and (**b**) release studies of ciprofloxacin of (■) *net*-PEG, (●) (*net*-PEG)-*g*-HEMA_30%_, and (▲) (*net*-PEG)-*g*-HEMA_66%_. *n* = 3.

Figure 5a shows that *net*-PEG was able to retain 1.5 mg per gram of material, while (*net*-PEG)-*g*-HEMA increased the loading to 2 mg g^−1^ and it was independent of the grafted HEMA amount. Therefore, the amount of HEMA in comb-like hydrogel did not significantly influence drug loading. These results are probably due to HEMA containing hydroxyl (OH) side groups attached to the polymer backbone, which are those that interact with ciprofloxacin through hydrogen bonds. However, OH groups are not totally available because the chains could be coiled and cover them. This is more likely to happen when the chains are longer, i.e., with high graft percentages.

A different behavior was observed in vitro release studies (pH: 7.4 PBS, 37 °C) since drug delivery depended on HEMA grafting (Figure 5b). *net*-PEG released 56% of ciprofloxacin in the first 60 min and then this percentage gradually reached 83% at 24 h. Comb-like hydrogel (*net*-PEG)-*g*-HEMA_66%_ was able to release 38% of the loaded drug, in comparison 25% released by (*net*-PEG)-*g*-HEMA_30%_, at 60 min. Subsequently, both hydrogels delivered the drug slower until reaching 48 and 38% at 24 h, respectively.

Comb-like hydrogel releases drug slower than *net*-PEG because it has a more compact structure, since grafted chains reduced the pore size [37,39]. This also causes ciprofloxacin to be retained more superficially when the HEMA graft increases, preventing deeper penetration of the drug into pores of comb-like hydrogel. These findings suggest that (*net*-PEG)-*g*-HEMA has potential application as a controlled drug release system. Moreover, it can be used as antimicrobial material because it can retain the antibiotic in its structure, preventing bacterial contamination over long periods. In order to analyze the release mechanism of ciprofloxacin, the cumulative data were fitted to common kinetics models by Equations (1)–(4).

(1)Zero order     Mt=M0+k0t(2)First order     Mt=M0e−k1t(3)Higuchi     Mt=kHt0.5(4)Korsmeyer–Peppas MtM∞=kptn

where *M*_0_, *M_t_*, and *M*_∞_ are the initial amount of drug, the released drug at time (t), and infinitive time, respectively. The constants associated with each model are *k*_0_, *k*_1_, *k_H_*, and *k_p_*. A correlation coefficient (R^2^) was used to evaluate the best fit.

As shown in Table 1, *net*-PEG and (*net*-PEG)-*g*-HEMA displayed a good fit for Higuchi and Korsmeyer–Peppas models, suggesting a drug release mechanism based mainly on Fickian diffusion driven by a concentration gradient in the polymer matrix. This means that the release rate gradually decreases over time as the drug migrates through the polymer matrix. The Korsmeyer–Peppas release exponent (n) further corroborates this mechanism because all samples exhibited values < 0.5. On the other side, the *k_H_* and *k_p_* parameters of *net*-PEG are higher than those of the comb-like hydrogel, indicating a faster diffusion-controlled drug release. Thus, HEMA grafting was a crucial factor in controlling ciprofloxacin delivery.

#### 2.3.4. Antimicrobial Activity

**Figure 6:**
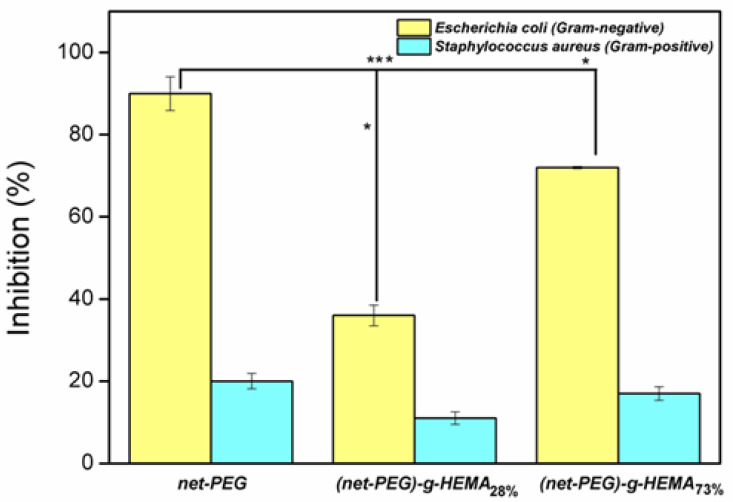
Inhibition of *E. coli* and *S. aureus* in hydrogels loaded with ciprofloxacin. * Indicates statistical significance (*p* < 0.05). *** *p* < 0.001 vs. control (*net*-PEG).

Antimicrobial studies with Gram-negative (*Escherichia coli*) and Gram-positive (*Staphylococcus aureus*) bacteria showed that hydrogels without ciprofloxacin did not present bacterial inhibition in *net*-PEG and (*net*-PEG)-*g*-HEMA with 28 and 73% grafting (data are not displayed), although both are reported in the literature as antibiofouling polymers [42,43]. However, hydrogels loaded with the antimicrobial drug showed different effects (Figure 6).

For the Gram-negative bacterium, *net*-PEG showed a bacterial inhibition of 90% which was attributed to the high swelling in the aqueous medium and its great capacity for diffusion and incorporation of the antimicrobial drug in the PEG polymeric network [44,45]. Comb-like hydrogel exhibited a smaller inhibition effect than *net*-PEG, displaying an inhibition of 72% for (*net*-PEG)-*g*-HEMA_73%_ and 36% for (*net*-PEG)-*g*-HEMA_28%_. Similar behavior was observed for the Gram-positive bacterium, where *net*-PEG showed the highest bacterial inhibition (20%), while comb-like hydrogels (*net*-PEG)-*g*-HEMA_28%_ and (*net*-PEG)-*g*-HEMA_73%_ displayed 11 and 17%, respectively. The low inhibition of ciprofloxacin in Gram-positive bacteria has already been reported in the literature; these bacteria have thick cell walls of peptidoglycan which limits the permeability to fluoroquinolone drugs such as ciprofloxacin [46,47].

The minimum inhibitory concentration (MIC) of ciprofloxacin for *S. aureus* is 0.015–0.03 μg mL^−1^, while for *E. coli* it is 0.125–0.5 μg mL^−1^. These findings are in agreement with those observed in the drug release study, where *net*-PEG and comb-like hydrogels (30 and 66% HEMA grafting) delivered 2.06, 1.266, and 1.6 μg mL^−1^ at 24 h, respectively (Figure 5b). This indicates that free ciprofloxacin is above the MIC for both bacteria. *net*-PEG showed better bacterial inhibition since it was able to release more antimicrobial drug than comb-like hydrogel, even though it loaded less drug. On the other hand, the ciprofloxacin liberation was dependent on grafted HEMA. (*net*-PEG)-*g*-HEMA_73%_ showed better inhibition than (*net*-PEG)-*g*-HEMA_28%_ for both bacteria because the drug release was higher as shown by the results of release kinetics models (Table 1).

According to these findings, comb-like hydrogel (*net*-PEG)-*g*-HEMA loads a larger amount of ciprofloxacin but releases less drug than *net*-PEG. Thus, a new slow-release material was obtained that has the possibility of preventing subsequent bacterial colonization, since it retains the ciprofloxacin inside. This behavior could be useful for chronic wounds which need delivery of an antibiotic to treat a local infection and keep the wound covered against future infections [48].

#### 2.3.5. Evaluation of Biocompatibility

**Figure 7:**
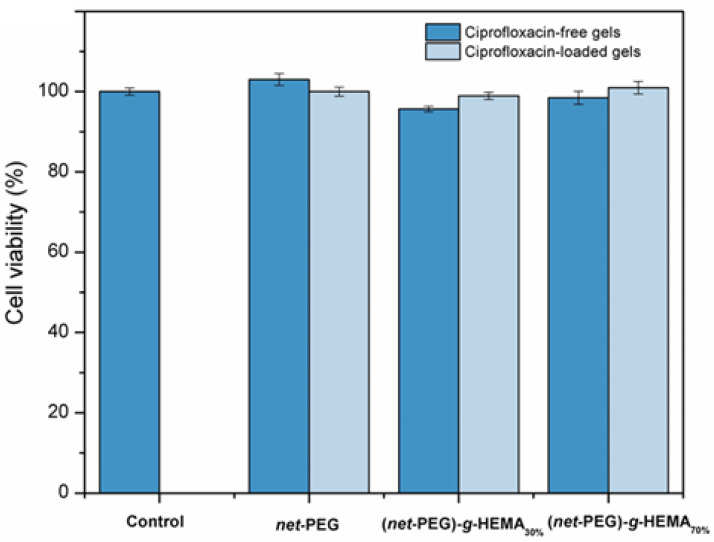
Cell viability of *net*-PEG and comb-like hydrogels using mouse embryonic fibroblasts (BALT/3T3). One-way ANOVA revealed no statistically significant differences among the groups (*p* > 0.05).

A crucial factor for biomaterials is biocompatibility, which ensures their safe use in living organisms. It essentially means that a biomaterial can perform its function in the body without causing adverse, systemic, or local effects [49]. In this case, a study of cell ability was performed using mouse embryonic fibroblasts (BALT/3T3) due to their rapid growth rates and the availability of standardized protocols. Results demonstrate that all hydrogels with and without antimicrobial drug loading (ciprofloxacin) present adequate biocompatibility properties (Figure 7).

In all cases, the materials showed a cell viability more than 95%. The results are attributed to the high inherent biocompatibility of both materials (PEG and HEMA), which has been reported in many research articles [50,51]. Furthermore, it demonstrates that the use of gamma radiation for modification of PEG hydrogel does not compromise its application as a biomaterial.

## 3. Conclusions

A comb-like hydrogel (*net*-PEG)-*g*-HEMA was successfully synthesized through a gamma radiation technique. The optimum conditions for the PEG crosslinking were reached using a concentration of 5% *w*/*v* and 30 kGy, while HEMA grafting was obtained using doses of 2–8 kGy. The HEMA incorporation was confirmed by FTIR and TGA. Swelling kinetics, ciprofloxacin loading and release, antimicrobial activity, and biocompatibility assays displayed interesting results depending on the amount of HEMA grafting.

Although water absorption decreased as grafting increased, ciprofloxacin loading (2 mg g^−1^) was not affected by this factor. However, an opposite behavior was observed in the delivery study where drug release was influenced by the presence of HEMA. *net*-PEG released 2.06 μg mL^−1^, while (*net*-PEG)-*g*-HEMA with 30% and 66% grafting were able to release 1.266 and 1.6 μg mL^−1^, respectively. These results are according to antimicrobial studies performed using *E. coli* (Gram-positive) and *S. aureus* (Gram-negative), where *net*-PEG displayed better antimicrobial activity than comb-like hydrogel for both bacteria as PEG hydrogel released more ciprofloxacin. Nevertheless, the inhibition of Gram-negative bacteria was superior in all hydrogels (35–90% inhibition). Notably, *net*-PEG and (*net*-PEG)-*g*-HEMA without ciprofloxacin did not present bacterial inhibition, although both are reported in the literature as antibiofouling polymers. In addition, all hydrogels displayed a cell viability of more than 95% using mouse embryonic fibroblasts (BALT/T3), proving that they are biocompatible materials.

According to these findings, the comb-like hydrogel could be used as antimicrobial material because it can retain the antibiotic in its structure, preventing bacterial contamination over long periods. In addition, other potential applications in the biomedical field could be wound dressings or drug delivery systems, but first it is necessary to address the limitations of this study which include the determination of water vapor permeability, mechanical stability, morphological studies, and in vivo performance.

## 4. Materials and Methods

PEG (Mw: 20,000), HEMA, and ciprofloxacin were purchased from Sigma-Aldrich, Toluca, Mexico. HEMA was vacuum distilled before use. Ethanol (Reproquim, Jalisco, Mexico) and distilled water were supplied by local companies. A ^60^Co radiation source (Gammabeam 650 PT, Nordion, Ottawa, ON, Canada) of 60,000 Ci was utilized for the crosslinking and grafting reactions.

### 4.1. Crosslinking of Poly(ethylene glycol)

PEG aqueous solutions (5 mL) at concentrations from 2.5 to 15% *w*/*v* were placed in Pyrex ampoules and bubbled with argon for 15 min to eliminate the oxygen present, sealed, and irradiated at 30 and 50 kGy using a dose rate of 6.89 kGy h^−1^. After irradiation, the ampoules were broken and *net*-PEG was stirred with water for 48 h and ethanol for 12 h to eliminate the monomer and un-grafted homopolymer, vacuum dried, and weighed. The crosslinking percentage was determined by Equation (5).

(5) $$Crosslinking\;\left(\%\right)=\frac{W_{f}}{W_{i}}\times 100\;\;$$

where *W_f_* and *W_i_* are the final and initial PEG weight, respectively.

### 4.2. Synthesis of Comb-like Hydrogel

This material was synthesized by grafting HEMA onto PEG hydrogel by a gamma radiation technique. *net*-PEG samples (200 mg) were placed in Pyrex ampoules with 4 mL of HEMA solution (5 and 10% *v*/*w*) using EtOH:H_2_O (1:1) as solvent, then they were bubbled with argon for 15 min to eliminate oxygen, sealed, and irradiated at doses from 2 to 15 kGy with a dose rate of 6.89 kGy h^−1^. After irradiation, comb-like hydrogels (*net*-PEG)-*g*-HEMA were extracted, changing the solvent each 12 h, and treated with ethanol for 24 h. Then, samples were dried in a vacuum oven at 30 °C until they reached constant weight. The grafting percentage was determined by Equation (6).

(6) $$Graft\;\left(\%\right)=\frac{W_{f}-W_{i}}{W_{i}}\times 100$$

where *W_f_* and *W_i_* are the final and initial weight of *net*-PEG, respectively.

### 4.3. Characterization Techniques

Crosslinked PEG and comb-like hydrogel were characterized by FTIR-ATR and TGA to confirm the HEMA incorporation. The hydrophilicity, antimicrobial properties, and cell viability were observed by swelling studies and biological assays, respectively.

FTIR-ATR: The attenuated total reflectance Fourier transform infrared spectrum was determined in a Perkin-Elmer Spectrum 100 spectrometer (Norwalk, CA, USA) with 16 scans in the range of 4000 to 650 cm^−1^.

TGA: For thermogravimetric analysis a TGA Q50 TA instrument was used (New Castle, DE, USA), with a heating rate of 10 °C min^−1^ from ambient temperature to 800 °C and a nitrogen atmosphere.

Swelling kinetics: Dried samples were weighed and immersed in receptacles with water at room temperature (25 °C) for different periods of time. Then samples were removed from the water, the superficial water was eliminated with towel paper, and the samples were weighed again and returned to the receptacles. This process was carried out until reaching a constant weight. Swelling percentage was calculated with Equation (7).

(7) $$Sw\;\left(\%\right)=\frac{W_{sw}-W_{d}}{W_{d}}\times 100$$

where *W_sw_* and *W_d_* are the swollen and dry weight of samples at any time.

Loading and release of ciprofloxacin: Ciprofloxacin was used as a drug model because it is a broad-spectrum antibiotic commonly used to treat many bacterial infections. *net*-PEG and (*net*-PEG)-*g*-HEMA (50 mg) were immersed in 10 mL of an aqueous solution of the drug (10 μg mL^−1^) and magnetic stirring (40 rpm) was carried out at 25 °C. Small aliquots were taken at different time intervals, measuring the change of absorbance by UV spectroscopy at 265 nm (UV-Vis spectrometer SPECORD200 Plus, Analytic Jena, Jena, Germany) and subsequently returning them to the receptacles. After 24 h, the samples were removed from the drug solutions and gently rinsed with water and the excess was dried with a paper towel. Then they were dried in a vacuum oven for 24 h at room temperature. Dried loaded samples were placed in 10 mL of buffer saline solution (PBS, pH: 7.4) at 37 °C with magnetic stirring (40 rpm). The absorbance was also monitored at different time intervals by UV-Vis spectroscopy at 265 nm. The experiments of loading and release were carried out in triplicate with independent samples.

Antimicrobial assay: Before the study, *net*-PEG and comb-like hydrogels with 28 and 73% HEMA graft were cut into discs (5 mg). The hydrogels were disinfected with a 90% hydroalcoholic ethanol dilution and dried at room temperature. Samples with and without antimicrobial drug were tested. Ciprofloxacin was loaded into samples by immersing them for 24 h in 10 mL of drug aqueous solution (10 µg mL^−1^). The gels were removed and dried at room temperature to begin the release and antimicrobial activity tests.

Brain–heart broth (BHI, Becton Dickinson, Franklin Lakes, NJ, USA) was prepared in sterile 5 mL tubes (3 mL/tube), and BHI agar was prepared in Petri dishes according to the manufacturer’s specifications. Media were autoclaved (121 °C, 15 min, 15 psi) and stored at 4 °C until use. Reference strains, *Escherichia coli* ATCC 51813 and *Staphylococcus aureus* ATCC 29213 (Microbiologics, St. Cloud, MN, USA), were reactivated by streaking on BHI agar and incubating at 37 °C ± 2 for 18–24 h in an aerobic atmosphere. Isolated colonies of each strain were suspended in sterile saline (0.85% NaCl, Sigma-Aldrich) and adjusted to the 0.5 McFarland turbidity standard (1.5 × 10^8^ CFU/mL) by spectrophotometry (Thermo Scientific™ Genesys™ UV-Vis Spectrophotometer, Waltham, MA, USA, λ: 625 nm). For each strain, 20 independent tubes were inoculated with sterile BHI (3 mL), each containing 100 µL of the standardized bacterial suspension (0.5 McFarland). The tubes were enriched with 5 mg of *net*-PEG, (*net*-PEG)-*g*-HEMA_28%_, and (*net*-PEG)-*g*-HEMA_73%_ and homogenized by vortexing (Vortex Mixer VWR^®^, Radnor, PA, USA, 10 s at 2500 rpm). As controls, tubes with BHI inoculated without additives were included. All assays were carried out in a centrifugation chamber. The bacteria were incubated under static conditions at 37 °C ± 2 (Memmert^®^ Incubator, Schwabach, Germany) for 24 h. Then, an aliquot (1 mL) was collected from each tube to measure the optical density. Growth inhibition was performed by measuring absorbance at a wavelength of 625 nm, comparing the treated tubes to the controls. Three independent biological replicates validated the results

Biocompatibility assay: A BALB/3T3 murine fibroblast cell line (ATCC CCL-163, Manassas, VA, USA) was used to evaluate the hydrogels’ cytocompatibility. Samples were cut into discs (20 mg) and sterilized as an antimicrobial assay. The gels were placed in Dulbecco’s modified Eagle’s medium (DMEM, Sigma Aldrich, USA) for 24 h. The incubated medium was then placed in contact with the fibroblasts for 24 h, and cell proliferation was subsequently assessed by reducing MTT to formazan (MTT kit, Roche, Basilea, Switzerland). Readings were taken using a Thermo Scientific Multiskan FC spectrophotometer at 620 nm. Cell viability was determined by comparing the absorbances obtained from the gels and the control (fibroblasts not in contact with the gels). Experiments were performed in triplicate with independent samples.

## Figures and Tables

**Table 1:** Ciprofloxacin release kinetics parameters.

Sample	Zero Order		First Order		Higuchi		Korsmeyer–Peppas		
	K_o_ (mg min^−1^)	R^2^	K_1_ (min^−1^)	R^2^	K_H_ (mg min^−0.5^)	R^2^	K_p_ (min^−1^)	n	R^2^
*net*-PEG	0.0247	0.8302	0.0004	0.7671	1.1451	0.9481	29.2146	0.1379	0.9014
(*net*-PEG)-*g*-HEMA_30%_	0.0112	0.9033	0.0003	0.8444	0.4842	0.9195	15.5561	0.1239	0.9302
(*net*-PEG)-*g*-HEMA_66%_	0.0126	0.7165	0.0003	0.6585	0.6158	0.9099	21.7121	0.1208	0.9666

## Data Availability

The original contributions presented in this study are included in the article. Further inquiries can be directed to the corresponding author.
